# Supplementary material for: Impact of an Educational Intervention for Healthy Eating in Older Adults: A Quasi-Experimental Study
Source: Int J Environ Res Public Health. 2023 Sep 25;20(19):6820. doi: 10.3390/ijerph20196820 (PMC10572856; doi:10.3390/ijerph20196820)
Supplement: Supplementary file 1 [file ijerph-20-06820-s001.zip › ijerph-2525731-supplementary.pdf]

Table S1. Demographic characteristics of the participants

	Control group (n = 58)	Intervention group (n = 51)	*t/X²/ Fisher's Exact*	*P*
	**N (%)**	**N (%)**		
**Age**				
Average	76.86	72.07		
Standard deviation	7.77	5.60		< 0.05
**Gender**				
Male	15 (25.86)	7 (13.73)		> 0.05
Female	43 (74.14)	44 (86.27)		
**Marital status**				
Single	9 (15.52)	5 (9.80)		
Married	30 (51.72)	32 (62.75)	> 0.05	
Widowed	16 (27.59)	9 (17.65)		
Divorced	3 (5.17)	5 (9.80)		
**Education level**				
Illiterate	21 (36.21)	12 (23.53)		> 0.05
Literate	37 (63.79)	39 (76.47)		
**Con quien vive**				
Living alone	19 (32.76)	11 (21.57)		> 0.05
Living with relatives	39 (67.24)	40 (78.43)		
**Economic stratum**				
High	0 (0.00)	3 (5.88)	> 0.05	
Medium and low	58 (100.00)	48 (94.12)		

Table S2. Bivariate analysis of the difference in knowledge, before and after the educational intervention and associated demographic characteristics.

	Before the educational intervention							After the educational intervention						
	**Group control**		**Intervention group**					**Group control**		**Intervention group**				
	**N = 58**	**(%)**	**N = 51**	**(%)**	**OR**	**IC 95%**	**P**	**N = 58**	**(%)**	**N = 51**	**(%)**	**OR**	**IC 95%**	**P**
≥ $\overline{X}$ Composite indicator	23	(39.66)	24	(47.03)	1.35	(0.63 - 2.90)	>0.05	17	29.31)	31	60.78)	3.74	(1.43-9.79)	< 0.05
**Demographic Variables**														
**Age group**														
65 a 74	5	(20.83)	17	(70.83)	5.30	(1.76 - 15.95)	< 0.05	0	(0.00)	21	(87.50)	-	-	-
75 a 84	10	(41.67)	6	(25.00)	0.64	(0.21 - 1.92)	> 0.05	10	(41.67)	9	(37.50)	1.03	(0.38-2.8)	> 0.05
80 y más	8	(80.00)	1	(10.00)	0.13	(0.02 - 1.04)	> 0.05	7	(70.00)	1	(10.00)	0.15	(0.02-1.23)	> 0.05
**Gender**														
Male	8	(53.33)	4	(26.67)	0.53	(0.15 - 1.89)	> 0.05	8	(53.33)	5	(33.33)	0.68	(0.21-2.23)	> 0.05
Female	15	(34.88)	20	(46.51)	1.85	(0.78 - 4.37)	> 0.05	9	(20.93)	26	(60.47)	5.66	(2.2-14.59)	< 0.001
**Civil status**														
Single	3	(33.33)	2	(22.22)	0.75	(0.12 - 4.67)	> 0.05	1	(11.11)	2	(22.22)	2.33	(0.2-26.46)	> 0.05
Married	15	(50.00)	16	(53.33)	1.31	(0.55 - 3.11)	> 0.05	11	(36.67)	19	(63.33)	2.54	(1.03-6.24)	
Widower	5	(31.25)	5	(31.25)	1.15	(0.31 - 4.24)	> 0.05	4	(25.00)	7	(43.75)	2.15	(0.59-7.85)	> 0.05
Divorced	0	(0.00)	1	(33.33)	-	-	> 0.05	1	(33.33)	3	-	3.56	(0.36-35.4)	> 0.05
**Educational level**														
Illiterate	9	(42.86)	4	(19.05)	0.46	(0.13 - 1.61)	> 0.05	5	(23.81)	10	(47.62)	2.59	(0.81-8.21)	> 0.05
Elementary	13	(36.11)	15	(41.67)	1.44	(0.59 - 3.5)	> 0.05	12	(33.33)	15	(41.67)	1.60	(0.65-3.92)	> 0.05
High school and	1	(100.00)	5	(500.00)				0	(0.00)	6	-			
university					6.20	(0.7 - 55)	> 0.05					-	-	-
**Who does he live with**														
Alone	10	(52.63)	4	(21.05)	0.41	(0.12 - 1.4)	> 0.05	5	(26.32)	7	(36.84)	1.69	(0.5-5.71)	> 0.05
With husband / wife	9	(42.86)	10	(47.62)	1.33	(0.49 - 3.62)	> 0.05	7	(33.33)	10	(47.62)	1.78	(0.62-5.12)	> 0.05
With other relatives	4	(22.22)	10	(55.56)	3.29	(0.96 - 11.32)	> 0.05	5	(27.78)	14	(77.78)	4.01	(1.31-12.24)	< 0.05
**Economic stratum**														
High	0	-	2	-	-	-	> 0.05	0	#¡DIV/0!	1	-	-	-	
Medium	9	(28.13)	21	(65.63)				9	(28.13)	27	(84.38)			
					3.81	(1.5 - 9.71)	< 0.05					6.13	(2.37-15.85)	< 0.001
Low	14	(53.85)	1	(3.85)				8	(30.77)	3	(11.54)			
					0.06	(0.01 - 0.5)	< 0.05					0.39	(0.1-1.56)	

≥ $\overline{X}$ *Composite indicator: It was obtained by statistically weighting the responses of the participants using the Principal Component Analysis technique. OR: Odds ratio. CI: Confidence interval. p: p-value.*

Table S3. Logistic regression of demographic characteristics associated with knowledge about healthy eating, after the educational intervention.

Variables (Reference)	B	S.E.	Wald	P	OR	C.I. 95%
**Age (65 a 74)**						
75 a 84	-1.990	0.858	5.382	0.020	0.137	(0.025-0.734)
85 and more	-1.101	0.821	1.798	0.180	0.332	(0.066-1.663)
**Gender (Male)**						
Female	0.402	0.571	0.497	0.481	1.495	(0.489-4.577)
**Marital status (Married)**						
Single	-1.786	1.123	2.527	0.112	0.168	(0.019-1.516)
Widower	0.132	0.858	0.024	0.878	1.141	(0.212-6.133)
Divorced	-0.614	0.900	0.466	0.495	0.541	(0.093-3.157)
**Educational level (Elementary)**						
Illiterate	-1.140	1.010	1.274	0.259	0.320	(0.044-2.316)
High school and university	-1.614	0.912	3.136	0.077	0.199	(0.033-1.188)
**Who do you live with (Alone)**						
With husband/wife	0.201	0.652	0.096	0.757	1.223	(0.341-4.386)
With other relatives	-0.462	.586	0.620	0.431	0.630	(0.2-1.989)
**Economic level (High)**						
Medium	0.087	1.569	0.003	0.956	1.091	(0.05-23.654)
Low	0.815	0.591	1.903	0.168	2.260	(0.71-7.199)

B: Beta Coefficient. S.E: Standard Error. Wald: Wald's Chi-square test. p: p-value. OR: Odds ratio. CI: Confidence interval.
